# Systematic Mapping Literature Review of Mobile Robotics Competitions

**DOI:** 10.3390/s22062160

**Published:** 2022-03-10

**Authors:** Laiany Brancalião, José Gonçalves, Miguel Á. Conde, Paulo Costa

**Affiliations:** 1 Polytechnic Institute of Bragança, 5300-253 Bragança, Portugal; laiany@ipb.pt; 2 Research Centre in Digitalization and Intelligent Robotics (CeDRI), 5300-253 Bragança, Portugal; 3 Robotics Group, Engineering School, University of Léon, Campus de Vegazana s/n, 24071 Léon, Spain; mcong@unileon.es; 4 Institute for Systems and Computer Engineering (INESC TEC), 4200-465 Porto, Portugal; paco@fe.up.pt; 5 Faculty of Engineering, University of Porto, 4200-465 Porto, Portugal

**Keywords:** robotic competitions, challenges, evaluation, benchmarking, robotic applications

## Abstract

This paper presents a systematic mapping literature review about the mobile robotics competitions that took place over the last few decades in order to obtain an overview of the main objectives, target public, challenges, technologies used and final application area to show how these competitions have been contributing to education. In the review we found 673 papers from 5 different databases and at the end of the process, 75 papers were classified to extract all the relevant information using the Preferred Reporting Items for Systematic Reviews and Meta-Analyses (PRISMA) method. More than 50 mobile robotics competitions were found and it was possible to analyze most of the competitions in detail in order to answer the research questions, finding the main goals, target public, challenges, technologies and application area, mainly in education.

## 1. Introduction

Robotics technology is increasingly present in our daily life and even more in industry. Inside this context, the emergence of robotics competitions around the world has provided great benefits for society. Robotics competitions are an excellent tool for the development of new solutions and innovations, to push the state of the art in several fields, benchmarking and even to motivate students to participate in science, technology, engineering and mathematics (STEM) areas and to encourage them to join engineering careers [1,2].

The concept of robotics competitions begins in 1977 when IEEE Spectrum magazine had the intention to make an Amazing Micromouse Competition. The first event took place in New York in 1979 where the goal was for a mobile robot to complete a maze as fast as possible. Later, Micromouse became very popular in Europe, Japan and USA until nowadays. Due to the success of Micromouse, Dean Kamen founded FIRST (For Inspiration and Recognition of Science and Technology) Association in 1989 and then the FIRST Robotic Competition season happened in 1992 in which high school students had to build and program a robot to complete a challenge [3,4].

Attached to the growth of the digital world other robotics competitions were being created, including different types of robots, themes, challenges and scenarios. Currently, it is possible to find many competitions related to mobile robots like humanoid robots, automated guided vehicles (AGV), unmanned aerial vehicles (UAV) and even underwater robots. The themes and scenarios can vary from rescue, dance, domestic service, logistics and manufacturing, marine services, virtual robots to soccer games. The challenges range from the simplest to the most complex and the goals facing industry, domestic tasks, education, natural disaster and benchmarking [4].

Among the types of robots and robotics competitions, the most common robots found are the mobile robots which are growing over the last years. Mobile robots applications have been widely implemented in industry and even in the domestic context. For some industrial tasks like transportation from one place to another, the AGVs can be useful once they are able to move in a dynamic environment with unexpected obstacles [5]. The service robots, which are mobile robots too, are designed for domestic tasks and are useful to assist people with disabilities. A famous competition related to this theme is RoboCup@Home, which started in 2006 as a new league of the RoboCup competition, which includes many other leagues [6]. The autonomous navigation of mobile robots also contributes to applications like autonomous cars [7].

According to advances in robotics and the contributions that robotics competitions have been providing, these competitions have been gaining attention in the education area, as a way to encourage students to STEM concepts, attracting them to pursue a career in the fields of technologies, promote the values of the engineering profession and also assist in teaching several multidisciplinary engineering topics and disciplines at universities [8,9]. Some of the most popular robotics competitions with a focus on education is the FIRST Robotics Competition, BotBall and RoboCupJunior [10].

The objective of this work is to present a systematic mapping literature review about the mobile robotics competitions which took place over the last few decades. The intention is to find many topics related to each competition like the target public and its age, the main places where the competitions take place, the different types of challenges, technologies applied and final application area. Finally, it is intended to have an overview of all the types of mobile robotics competitions with detailed descriptions, the different goals, the results that have been found and how the competitions can contribute positively to education.

This paper is structured as follows: Section 2 explains the systematic mapping literature review process and describes the planning done for this theme and how to conduct the review. Section 3 shows all the numbers related to the papers found and details the conducting process of the review that was done. Section 4 presents, in a detailed form, all the mobile robot competitions found and discussed the answers to the research questions. Finally, Section 5 ends our review.

## 2. Methodology

This paper followed the systematic mapping literature review methodology, also called literature mapping, which is useful at the beginning of research for the contextualization of ideas. literature mapping aims to seek all the knowledge available about an idea and find, at the end of the survey, the most relevant papers according to your research questions. It is also used to complement a systematic literature review (SLR), which is another methodology of evaluating all available research and evaluating the relevant papers related to the main idea. The difference between SLR and literature mapping is that the last one is wide and SLR is more specific, but when the both are used together the best results are found. Commonly, first one develops literature mapping to have an overview of the theme and then an SLR is obtained in order to obtain more specific and detailed results [11,12,13,14,15].

The literature mapping process follows some steps which are practically the same as the SLR steps that will be described below. Before starting any SLR or literature mapping, the first step is to make a search on the Internet in order to verify if there already is a literature mapping about the intended topic. If there is a literature mapping or even an SLR, it is not necessary to conduct another one, however, if no results were found for the specific idea selected, the literature mapping or SLR can be implemented following the steps of the planning and conducting described below [13,14].

### 2.1. Planning

#### 2.1.1. Planning the Research Questions

The first step to start the systematic mapping literature review is to elaborate the research questions, which are focused on a theme and the points to be discovered, understood or studied must be found in its answers. These questions must define clearly the problem to be solved. It is important to emphasize that the research questions of mapping are broader than those created for the SLR [11]. Taking into account that the main context of this work is mobile robotics competitions, the research questions are:

- **RQ1:** What type of mobile robotics competitions exist in the last few decades and with what aim?
- **RQ2:** Where do the mobile robotics competitions take place currently who and is their target public?
- **RQ3:** What type of robotics challenges are addressed by the mobile robotics competitions?
- **RQ4:** What type of technologies are used in mobile robotics competitions?
- **RQ5:** What is the final application area of the mobile robotics competitions?
- **RQ6:** How have these competitions been contributing positively to education?

#### 2.1.2. Elaborating the PICOC

Once the research questions are made, the next step is to perform the PICOC method proposed by Petticrew and Roberts [12], which assists in the article analysis process. The description about each topic is presented below:

- Population (P): Who?
- Intervention (I): What and How?
- Comparison (C): What to compare?
- Outcome (O): The final objectives, what does the search obtain or improve?
- Context (C): What are the circumstances?

The scope chosen for this work was as follows:

- Population (P): mobile robotics competitions;
- Intervention (I): detailed description about mobile robotics competitions;
- Comparison (C): compare all the mobile robotics competitions;
- Outcome (O): types, goals, target public, challenges, technologies used and application areas of the mobile robotics competitions;
- Context (C): mobile robots.

#### 2.1.3. Selecting the Keywords and Synonyms

The keywords and synonyms will help to obtain the search string, which will be discussed in the next section and it is related to each PICOC item. According to the theme of this work, the keywords and their synonyms chosen are presented in Table 1.

#### 2.1.4. Inclusion and Exclusion Criteria

The inclusion and exclusion criteria help to define the relevant papers for the study and which might answer the research questions. A paper which presents all the inclusion topics can be relevant, but if it includes one or more exclusion topics this paper must be excluded.

Inclusion criteria:

- The work is written in English;
- The work was published after 2001;
- The work must have information about one or more robotics competitions;
- The work must have included the “robotic competition” term.

Exclusion criteria:

- The paper is not accessible;
- The work is not written in English;
- The work was published before 2001;
- Work does not involve a robotic competition context;
- Works that include the term “robotic competition” but does not answer any research question.

These criteria were chosen based on the fact that the first robotics competitions started to gain space in the 1990s, and even if there were some competitions that have already been created before, we chose to start some years later in order to ensure that concrete research and results could be collected [4].

#### 2.1.5. Creating the Search String and Choosing the Sources

After all the steps before it is possible to select the databases and create the search string easily. The sources chosen to search for the papers were ACM Digital Library, IEEE, Scopus, Springer Link and Web of Science because they are important repositories for research about technology.

The search string is also called a query and is an equation that represents all the main terms of the search. This string needs to be put on each database chosen to search for papers related to the theme, but depending on the website the search string varies and needs some specific characters.

The search string created for this work was:

(“robotics competitions” OR “robotic competition”) AND (“benchmark” OR “challenges” OR “challenge” OR “evaluation” OR “performance” OR “robotics application” OR “technologies” OR “technology” OR “validation”)

- ACM Digital Library: on the website we used the advanced search and the term “All” for each term in order to find it in any place of the paper. The query used in this data base was:
  [[All: “robotics competitions”] OR [All: “robotic competition”]] AND [[All: “performance”] OR [All: “challenges”] OR [All: “challenge”] OR [All: “robotics application”] OR [All: “technologies”] OR [All: “technology”] OR [All: “validation”] OR [All: “evaluation”] OR [All: “benchmark”]]
- IEEE: we used the same main equation shown at the beginning and added some terms like “Abstract”, “Author Keywords” and “Title” in the same place as “All”. This way we searched for the terms only in these topics of the paper;
- Scopus: on the website we used the same main equation in the advanced search tab and just added the term “TITLE-ABS-KEY” in the query, indicating that the search for the words is done only on the title, abstract and keywords of the paper. The modified query was:
  TITLE-ABS-KEY ((“robotic competition” OR “robotics competitions”)) AND TITLE-ABS-KEY ((“performance” OR “challenges” OR “challenge” OR “robotics application” OR “technologies” OR “technology” OR “validation” OR “evaluation” OR “benchmark”))
- Springer Link: exactly the same query cited at the beginning was used on the website simple search;
- Web of Science: the query was put in the search tab of the website and we added some terms at the beginning of the equation like “TI”, “AB” and “AK” indicating a specific search as explained before.
  TI = ((“robotics competitions” OR “robotic competition”) AND (“benchmark” OR “challenges” OR “challenge” OR “evaluation” OR “performance” OR “robotics application” OR “technologies” OR “technology” OR “validation”)) OR AB = ((“robotics competitions” OR “robotic competition”) AND (“benchmark” OR “challenges” OR “challenge” OR “evaluation” OR “performance” OR “robotics application” OR “technologies” OR “technology” OR “validation”)) OR AK = ((“robotics competitions” OR “robotic competition”) AND (“benchmark” OR “challenges” OR “challenge” OR “evaluation” OR “performance” OR “robotics application” OR “technologies” OR “technology” OR “validation”)).

#### 2.1.6. Quality Assessment Checklist

New questions are defined in this phase in order to verify the quality of a paper when it is read completely and before putting it in the final review. These questions can be more specific and each one has a weight, the quality questions elaborated for this work are presented below.

- **QQ1:** Is the paper based on research and not on expert opinion?
- **QQ2:** Is there a clear objective of the research?
- **QQ3:** Are the work results discussed well?
- **QQ4:** Is the work based on one or more robotics competitions?
- **QQ5:** If the work is based on one or more robotics competitions, is the competition described well?
- **QQ6:** Does the work describe the challenges and activities of the robotics competition?
- **QQ7:** Does the work present the new technologies applied to robotics competitions?
- **QQ8:** Does the work discuss the robotics competitions’ contribution to education or industry?
- **QQ9:** Does the information or data obtained by the work answer at least one of the research questions?

The answer value can be three values: 1.0 (if it answers the question fully), 0.5 (if it answers the question partially) or 0 (if it does not answer the question). Each paper can be evaluated with a maximum score of 9.0 and the cutoff score selected was 6.0 based on the most important questions of the list that needs to be answered fully, these were questions 4, 5, 6, 7, 8 and 9. These questions are more important than the three first because they are focused on the topics that we want to discover and are based on the research questions. Therefore, all the papers that exceed the score of 6.0 are included in the final review.

#### 2.1.7. Data Extraction Form

Once we already have all the relevant paper for the research, the last step is to apply the data extraction form, in this phase a new set of questions is created to extract all the important information of the final articles in order to assist to answer the research questions set at the beginning of the mapping. The data extraction questions selected for this work are:

- **DQ1:** What are the main robotics competitions taking place currently and their goals?
- **DQ2:** Where do the main robotics competitions take place?
- **DQ3:** Which ages does the robotics competitions cover?
- **DQ4:** What are the main challenges and activities at the robotics competitions?
- **DQ5:** What are the main technologies used in the robotics competitions?
- **DQ6:** Which application areas can robotics competitions contribute?
- **DQ7:** Which robotics competitions contributes positively to education?

### 2.2. Conducting

**Figure 1:**
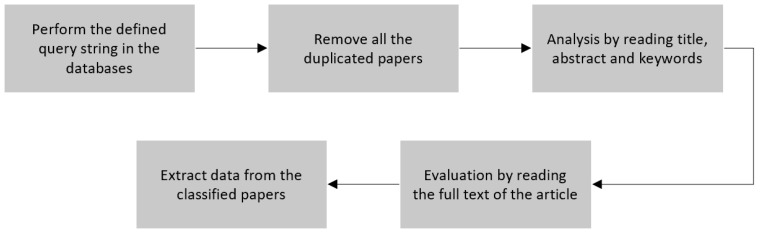
Conducting process. Source: Author.

After the planning stage is elaborated, the next step is to perform the conducting, which was done by following the PRISMA (Preferred Reporting Items for Systematic Review and Meta-Analysis) method that describes the phases of the conducting process [11]. The process is illustrated and exemplified in Figure 1.

- Identification: the papers found in each source using the query are saved and then the duplicate studies are removed.
- Screening: just the title, abstract and keywords are read applying the inclusion and exclusion criteria, the papers that are not approved by the criteria are removed too.
- Eligibility: for the remaining articles, we applied the quality questions, so the papers need to be read fully in order to obtain the answers for those questions and a good score. The papers that do not have a score above the limit must be deleted.
- Included: the papers with a high score are classified for the final review and we performed the data extraction using the data extraction form questions [11].

The tool used to perform this systematic mapping literature review was the Parsifal [16]. It is good to organize the steps, plan the review, import the papers, answer the questions and at the end generate a report about the review.

## 3. Results

This section presents the results obtained in the conducting process. In the identification stage we searched the databases using the query string and 673 papers were found in total, 63 from ACM Digital Library, 28 from IEEE, 300 from Scopus, 222 from Springer Link and 60 from Web of Science. There were 104 reports duplicates, removing them left 569. In the screening stage we performed the inclusion and exclusion criteria and of the remained articles, 242 passed to the next phase and 327 were excluded because they did not include one or more inclusion and exclusion criteria.

**Figure 2:**
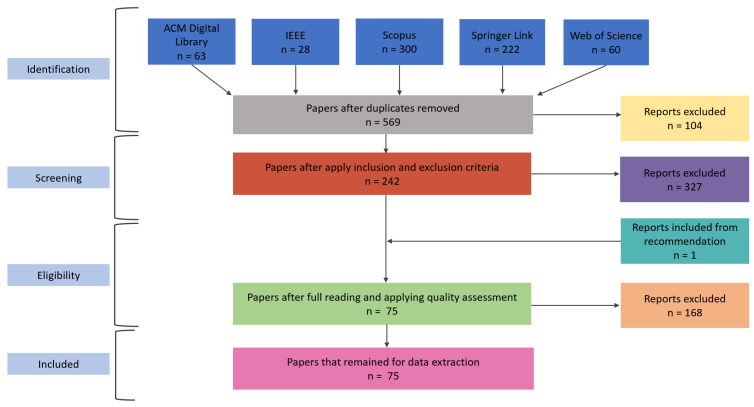
Results of conducting the review process. Source: Author.

In the eligibility stage, it is necessary to read all the articles fully in order to apply the quality assessment, then after this process, 168 papers were removed, leaving 74 papers in the included stage plus 1 which was a recommendation and which contained a mobile robotic competition that was not found in the chosen data sources, but was relevant for the research, totalling 75 papers. The articles classified for this last stage are those that will be used in the final review and which we will perform the data extraction on. All the conducting processes carried out are illustrated in the flowchart in Figure 2.

Table 2 shows all 75 papers that went through the quality assessment and were classified for the data extraction stage, the respective answers to the quality questions and the final scores, which were above 6.0. It is possible to notice that for question QQ4 most of the answers were yes, including just two partial answers, indicating that most of the articles were based on any robotic competition.

For question QQ9, all of the answers were yes, because all the papers answer at least one of the research questions before. The first two quality questions obtained good answers because the papers were well elaborated, including clear objectives and based on good research. Most of the QQ7 answers were no, because few articles really described the technologies used in the robotics competitions. For questions QQ3, QQ5, QQ6 and QQ8 the answer distribution was varied since some papers do not have a good results discussion, do not describe in detail the robotics competition which it is based on and do not present a competition application area.

**Figure 3:**
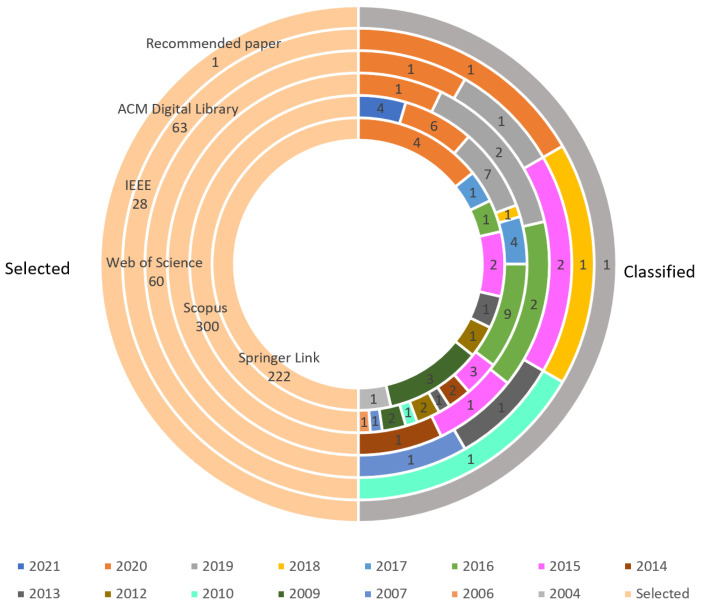
Selected and classified papers per source and year. Source: Author.

Figure 3 represents the number of papers selected per database on the left side and the number of papers classified per source and year on the right side. The selected articles are those obtained at the beginning of the review and the classified articles are those that remain at the end of the review, after applying the quality assessment and which will perform the data extraction.

It is possible to notice that Scopus and Springer Link were the databases in which a large number of articles were found and classified. The database which was found the lowest number of articles was IEEE but there were few classified articles were from ACM Digital Library. It is possible to see that most of the articles classified for the included stage were papers from 2020, 2019, 2016 and 2015, which represents recent data and results that have been added over years of competitions.

## 4. Discussion

After applying the data extraction phase in all the 75 papers we obtained enough information about all the robotics competitions found. Table 3 lists all the 67 robotics competitions found in total and the respective references. It is possible to notice that robotics competitions found in most of the papers were RoboCup and First Robotics Competition, which is famous competitions focused more on education. The DARPA Robotics Challenge was also cited in most articles, which includes autonomous vehicles and complex tasks [3,35,49,53,81].

**Figure 4:**
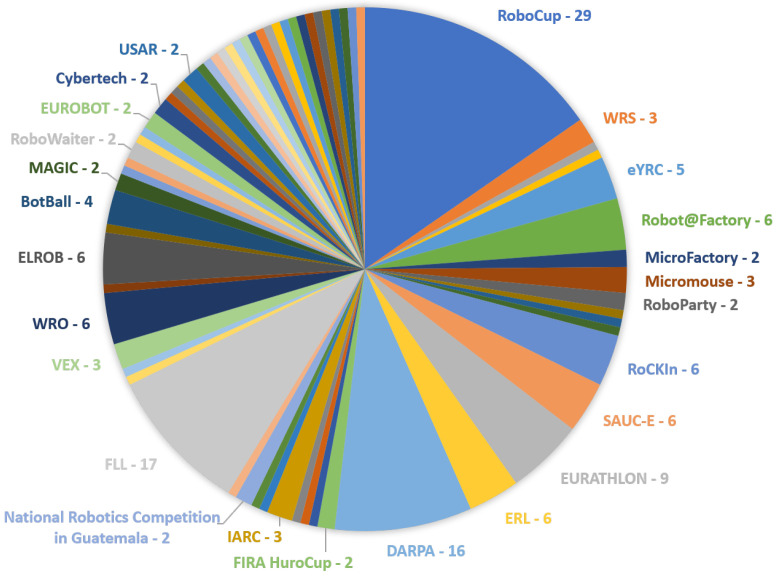
Robotics competitions per number of references in which they were found. Source: Author.

Figure 4 illustrates Table 3 and the numbers next to the competition name in the figure represent the number of articles in which the competition was cited. The spaces where the name is not specified are competitions cited in only one article. It is possible to see that the competitions most cited by the papers are RoboCup, FIRST and DARPA. The reason is probably that RoboCup is the biggest competition and one of the most famous, including many leagues in several domains, even football games with robots [49,81].

The FIRST is one of the oldest robotics competitions with a greater focus on education, attracting young students to careers in engineering and technology, besides that uses a well-known tool, LEGO kits [9,52]. The DARPA is a robotic competition that is more professional and industry-focused, focused on innovative solutions for problems and it has prize money. The first editions were focused on autonomous vehicles and then the other editions included humanoid robots [3,35,53].

Table 4 presents the relevant information about each robotics competition extracted from the last review stage and based on the data extraction questions presented in Section 2.1.7, each topic (second column) associated with each competition (first column) is related to the questions. It is noted that not all competitions listed in Table 3 are described in Table 4, because there are some competitions that were only cited in the papers, without detailed information. Therefore, in the table below are only the robotics competitions in which it was possible to obtain data through the selected papers, in total there were 38 competitions.

Table 5 presents all the mobile robotics competitions linked to the research concepts in which they are involved in each related paper. The proposal of this table is to provide the key researches associated with the competition’s challenges and the papers.

At the end of the review and after all the information is extracted, the research questions proposed in Section 2.1.1, in the beginning of the review, are finally answered. As Table 4 shows, there are a lot of robotic competitions around the world, since there are big and international competitions with several challenges and also simple competitions that are often done in a specific region or school. The following discussion is related to answering the research questions.

**RQ1: What type of mobile robotics competitions exist in the last few decades and with what aim?**

There are robotics competitions focused on education, that is, willing to teach robotics concepts to students, attracting them to STEM areas and to encourage them to enter in technologies careers. Most of the competitions found were focused on education like Robosub, Latin American IEEE Robotics Competition, RoboParty, Microfactory, Cybertech, EUROBOT, NIARC, Brazilian Robotics Olympiad, WRO, MSBEST, VEX, BRC, FIRST, RoboCup@Junior, Robot@Factory, eYRC and the “Schüler bauen Roboter” program [3,4,8,9,10,17,20,21,22,29,30,32,41,42,43,45,46,50,52,55,57,61,63,64,67,70,71,73,78].

A theme very commonly found in the robotics competitions was industry, like RoboCup Work and RoboCup Logistics League, WRS Assembly task, ARIAC, RoCKIn@Work and SICK robot day [37,38,48,59,75,76,77]. Robot@Factory and MicroFactory have a focus on education, but their themes are focused on industry and logistics too [41,46,63,67]. Other types of competitions found were those facing the domestic field, like RoboCup@Home, WRS Service Challenge, RoCKIn@Home and RoboWaiter, which their challenges usually are to perform house tasks in order to help elderly people or those with disabilities [6,25,28,37,75].

Some competitions found have sports as a theme and often have soccer games, like RoboCup@Soccer, FIRA HuroCup and WRO football category [4,24,81]. Besides that, there are competitions that include magic and dance like IEEE Humanoid Application Challenge [65], in which the robot needs to perform a magic show and RoboCup@Junior Dance Challenge [10,29] which the robot needs to perform a dance and its focus is more on education. There also are competitions totally online for example the CiberMouse@RTSS08 [23].

Many of the robotics competitions found have a focus on search and rescue based on natural disaster scenarios, including outdoor, indoor, terrain and even underwater environmental. Some examples are RoboCup Search and Rescue League, NIARC 2012 edition, EURATHLON, ERL and ERL Emergency, being the last three focused on the 2011 Fukushima accident. The NIARC is a competition that changes the theme every year, in the 2013 edition the theme was the mining industry and in 2014 was the agriculture industry. The EUROBOT competition follows the same way, changing its theme every year, in the 2010 edition the theme was collecting fruits and vegetables and the 2011 edition was a chess game [20,32,53,54,66,81].

There are also robotics competitions that are important tools to push the state of the art in a specific field, for example in the area of drones like MBZIRC and IARC [1,56]. HRATC contributes in the area of humanitarian demining and DARPA in autonomous vehicles [35]. SAUC-E, which can also cited, was the first underwater robotic competition in Europe and promotes advances in the state of the art of Autonomous Underwater Vehicle (AUV) [73].

**RQ2: Where do the mobile robotics competitions take place currently and who is their target public?**

Most of the international robotics competitions take place in many countries all over the world because it has an international scope [3,6,43,58]. Some educational competitions take place in universities or in a specific region, for example the “Schüler bauen Roboter” program that takes place in the University of Munich, Germany or Cybertech that take place in Universidad Politécnica de Madrid (UPM), Spain [21,57].

The target public of the competitions can vary a lot, but we can conclude that for the most part, it is students. The competitions focusing on education always include students and some other big, famous and international competitions have a larger target public including young students and senior participants, like engineers and business [3,6,43,58].

**RQ3: What type of robotics challenges are addressed by the mobile robotics competitions?**

The challenges addressed by the educational robotics competitions usually includes tasks that the participants have to build a robot to perform some activity, in order to allow them to apply the knowledge learned in classes in real-world problems and put it into practice. Sometimes there are several stages, like eYRC for example, in which the students firstly answer questions, realize a preliminary test or learn concepts about programming and electronics and then, they use tools provided by the competition to find the best solution for a problem, involving hardware and software approaches [21,29,42,45,50,57,71,78].

This kind of competition usually elaborates simple tasks like dance and games and with a focus on students working in teams and developing soft skills [3,10,29]. However, there also are competitions focused on education which includes industry challenges too, for example the Robot@Factory and MicroFactory [41,46,63,67].

In the robotics competitions focused on industry, the challenges can vary between transport products and logistics tasks, in which the robots must navigate, avoid obstacles and deliver products in correct positions, perform assembly tasks and kitting processes, build kits by picking up items, organize materials in warehouses, among others [37,38,41,46,48,59,63,67,75,76,77]. There also are challenges addressed to evaluate the robot speed, like activities in a maze (Micromouse) or delivering as many objects as possible (SICK robot day), in these kind of tasks the robots needs to be as fast as possible [3,38,60,70].

Home challenges are usually focused on the robot helping in household activities inside an environment that simulates a house including rooms. The tasks found in this kind of competition were organizing objects from an incorrect place to a correct place, assisting people in opening or closing windows and doors and kitchen activities like opening the refrigerator, picking up a plate of food from a shelf and putting it in a table [6,25,28,37,75].

The challenges included in search and rescue competitions can be exploration tasks, climbing ramps, walking on dirty terrain with poor visibility, looking for missing people, pipe inspection and stemming the leak, reconnaissance and environmental survey and usually, the scenarios simulate a natural disaster and it can vary between underwater, buildings, small places, indoor or outdoor environments [19,20,32,53,54,66,81].

In soccer games, the challenges usually include teams of autonomous robots competing against each other. FIRA Hurocup also includes other sports activities for the robots for example basketball, climbing wall, penalty kicks, lift and carry, weightlifting and obstacle run [4,24,81].

**RQ4: What type of technologies are used in the mobile robotics competitions?**

The main technologies used by the robotics competitions found in the papers were methods like AI solutions, machine learning, computer vision, simultaneous localization and mapping (SLAM), image processing, speech recognition, object recognition, gesture recognition, extended Kalman filters (EKF), real-time control and 3D printing. It was also found that there were a lot of software and robot simulators like ROS, HIL, SimTwo, Gazebo, TeamBots, USARSim, OpenCV, TensorFlow, LabView and also components like sensors, actuators, cameras, microcontrollers and LEGO Mindstorms kits [1,6,8,19,22,24,35,40,63,70,77,79].

**RQ5: What is the final application area of the mobile robotics competitions?**

In general, the mobile robotics competitions can contribute with advances in many fields, like industry, daily life, search and rescue in disaster scenarios, but one of the most benefited has been education.

Most of the competitions have been contributing to education recently, increasing the students’ interest in STEM concepts, introducing other people to the field of robotics, connecting students with professionals, enabling them to solve real world problems, encouraging them to join engineering careers and developing 21st-century skills. It is becoming more and more usual in schools and universities last few years because it aids them to teach a variety of multidisciplinary engineering topics including design, programming and mechatronics. Besides that it can also contribute to develop the ability of the teams to work together in a multidisciplinary work [3,4,8,9,10,17,20,21,22,29,30,32,41,42,43,45,46,50,52,55,57,61,63,64,67,70,71,73,78].

Another application area very common in robotics competitions is industry, contributing to the development of new solutions related to manufacturing and logistics, assisting people in repetitive tasks and reducing human errors. Besides that, the competitions have been a good way to promote comparison of different algorithms and systems and a means of discovering the best practices to solve real-world problems [37,38,41,46,48,59,63,67,75,76,77].

The competitions focused on home environments contributes to the development of solutions based on assistive robots, providing a benchmark for robot assistance, not only for disabled people but also for elderly people and healthy people, by assisting daily housekeeping tasks [6,25,28,37,75].

Search and rescue area can be benefited by the robotics competitions focused on this theme because encourages the development of solutions that allow autonomous robots to work and help in areas inaccessible for humans or in natural disasters areas [20,32,53,54,66,81].

There are some competitions that assist to push the state of the art in domains that are growing like air, land and sea, providing an opportunity to exchange ideas, create solutions as well as a venue to evaluate and encourage state of art research. Autonomous vehicles and drones are fields that have been attracting a lot of attention, mainly through robotics competitions [4,53,54,65,66].

**RQ6: How have these competitions been contributing positively to education?**

As we can see in Table 4 a large part of the competitions has a final application area in education. Associated with the last extracted form question DQ7 (Which robotics competitions contributes positively to education?) we listed most of the robotics competitions with a focus on education.

- RoboCup Junior;
- e-Yantra Robotics Competition (eYRC);
- Micromouse;
- Robot@Factory/Robot@Factory Lite;
- Balam Robot Competition (BRC);
- FIRST Robotics Competition/FIRST Lego League (FLL);
- VEX Robotics Competition;
- Student Autonomous Underwater Vehicle Challenge-Europe (SAUC-E);
- Mississippi BEST (MSBEST) robotics;
- World Robot Olympiad (WRO);
- Brazilian Robotics Olympiad;
- National Instruments Autonomous Robotics Competition (NIARC);
- EUROBOT competition;
- Cybertech;
- “Schüler bauen Roboter” program;
- MicroFactory;
- Roboparty;
- Latin American IEEE Robotics Competition;
- Robosub.

Most of them have as objective the dissemination of technology and STEM concepts through young students, encouraging them to pursue career in these fields, developing skills in programming, electronics, robotics, working in team, increasing the motivation towards engineering besides that assist universities to teach multidisciplinary domains [3,4,8,9,10,17,20,21,22,29,30,32,41,42,43,45,46,50,52,55,57,61,63,64,67,70,71,73,78]. The SAUC-E, for example, has one of the objectives like getting closer contact between the university teams and companies invited to participate [53].

The target public involves young students of primary and secondary schools, high school students and university and engineering students. A famous robotic competition called FIRST Lego League accept students from 9 to 14 years old, a lower age level than e-Yantra Robotics Competition for example, which includes college students of the Indian Institute of Technology Bombay [4,58,78].

Some competitions take place directly at universities like NIARC, which occurs in universities across Australia and New Zealand, Cybertech, which occur in Universidad Politécnica de Madrid, Balam Robot Competition (BRC) in Universidad Galileo and also “Schüler bauen Roboter” program in Technical University of Munich [21,32,57,61]. The other robotics competitions can take place in any other place or more than one like SAUC-E and EUROBOT competition, for example, which occur in many countries around Europe [20,30,62,73] or even RoboCup and FIRST which are competitions that take place all over the world [3,6,43,58].

FIRST Robotics Competition is one of the oldest and the most famous competition focused on education which began in 1998 with the FIRST Organization and the LEGO Group [52]. The most recent VEX robotics world championship run by the Robotics Education and Competition (REC) Foundation in April of 2018, became the “largest robot competition” in the world according to Guinness World Records [64].

Table 6 summarizes the research questions answers. On the left side there are the six research questions that this work aims to answer and on the right side are presented the main topics of each answer which were already discussed above but with fewer details, in order to provide better insights.

## 5. Conclusions

In this paper, a systematic mapping literature review was developed and it was found a large number of articles cited or/and described many mobile robotics competitions that took place over the last few decades. It was possible to analyze in detail most of the competitions and to conclude that these competitions are growing and becoming more common in several domains with diverse objectives, mainly in education. It was observed that the number of competitions since 2001 is gradually increasing each year.

Among the most cited robotics competitions in the articles are RoboCup, FIRST and DARPA. RoboCup is the biggest robotic competition with more than 10 leagues, covering several areas in a single competition but with different challenges. FIRST is one of the oldest robotics competitions, very famous too, and has a focus on education and each year a new theme is chosen. DARPA is a competition that is more professional and has prize money and is focused on autonomous vehicles.

It is possible to conclude that education is the area most benefited by the mobile robotics competitions. The number of competitions focused on contributing to education is growing because they have been provided how powerful they could be in attracting students for technological areas and positive results have been observed. The robotics competitions focused on education usually have objectives focused on encouraging young students to pursue careers in STEM areas, develop skills, teach how to work in team, assist teachers and universities in multidisciplinary domains and expose students to real problem solving and practical application of their knowledge.

Therefore, the robotics competitions have been a good contribution tool not only for education but for different areas, helping people, engineers, researchers, business and students to solve real problems through the use of robots and creating innovative solutions, showing that the robots can be assist us to construct a better quality of life for people and consequently a world better for all.

As future work, we intend to perform a systematic literature review, which is more specific than a mapping, of the mobile robotics competitions all over the world and the research questions could be more focused on education, industry or benchmarking.

## Figures and Tables

**Table 1:** Keywords and synonyms chosen for the review.

Keywords	Synonyms	Related to
Benchmark		Outcome
Challenges	Challenge	Outcome
Evaluation		Outcome
Performance		Outcome
Robotics application		Outcome
Robotics competitions	Robotic competition	Population
Technologies	Technology	Outcome
Validation		Outcome

**Table 2:** Results of quality assessment.

Reference	QQ1	QQ2	QQ3	QQ4	QQ5	QQ6	QQ7	QQ8	QQ9	Final Score
[1]	Yes	Yes	Yes	Yes	Yes	Yes	Yes	Partially	Yes	8.5
[2]	Yes	No	Partially	Yes	Yes	Yes	No	Yes	Yes	6.5
[3]	Yes	Yes	Yes	Partially	Partially	Partially	No	Yes	Yes	6.5
[4]	Yes	Yes	Yes	Yes	Yes	Yes	No	Yes	Yes	8.0
[5]	Yes	Yes	Yes	Yes	Partially	Partially	Partially	Partially	Yes	7.0
[6]	Partially	Yes	Yes	Yes	Yes	Yes	Yes	No	Yes	7.5
[7]	Yes	Yes	Yes	Yes	Partially	No	Partially	Partially	Yes	6.5
[8]	Yes	Yes	Yes	Yes	Yes	Yes	Yes	Yes	Yes	9.0
[9]	Yes	Yes	Yes	Yes	Yes	Yes	Partially	Yes	Yes	8.5
[10]	Partially	Yes	Partially	Yes	Yes	Yes	No	Yes	Yes	7.0
[17]	Partially	Yes	Yes	Yes	Partially	Yes	No	Yes	Yes	7.0
[18]	Yes	Yes	No	Yes	Yes	Yes	No	Yes	Yes	7.0
[19]	Yes	Yes	Partially	Yes	Yes	Yes	No	No	Yes	6.5
[20]	Yes	Yes	Yes	Yes	Yes	Yes	No	Yes	Yes	8.0
[21]	Yes	Yes	Yes	Yes	Yes	Yes	No	Yes	Yes	8.0
[22]	Yes	Yes	Partially	Yes	Yes	Yes	Yes	No	Yes	7.5
[23]	Yes	Yes	Yes	Yes	Yes	Yes	No	Yes	Yes	8.0
[24]	Yes	Yes	Partially	Yes	Partially	Partially	No	Yes	Yes	6.5
[25]	Yes	Yes	Partially	Yes	Yes	Yes	No	No	Yes	6.5
[26]	Yes	Yes	Partially	Yes	Yes	Partially	Yes	Partially	Yes	7.5
[27]	Yes	Yes	Yes	Yes	Yes	Yes	Partially	No	Yes	7.5
[28]	Yes	Yes	Partially	Yes	Yes	Yes	Partially	Yes	Yes	8.0
[29]	Yes	Yes	Partially	Yes	Yes	Yes	No	Yes	Yes	7.5
[30]	Yes	Yes	Yes	Yes	Yes	Partially	Partially	No	Yes	7.0
[31]	Yes	Yes	Yes	Yes	Yes	Yes	Yes	Partially	Yes	8.5
[32]	Yes	Yes	Yes	Yes	Partially	Partially	Yes	Partially	Yes	7.5
[33]	Partially	Yes	Partially	Yes	Yes	Yes	Partially	Yes	Yes	7.5
[34]	Yes	Yes	Yes	Yes	Yes	Yes	Yes	No	Yes	8.0
[35]	Yes	Yes	Yes	Yes	Yes	Yes	No	Yes	Yes	8.0
[36]	Yes	Partially	No	Yes	Yes	Yes	Partially	Partially	Yes	6.5
[37]	Yes	Partially	Yes	Yes	Yes	Partially	No	Partially	Yes	6.5
[38]	Yes	Yes	Partially	Yes	Yes	Yes	Partially	Yes	Yes	8.0
[39]	Yes	Yes	Yes	Partially	Yes	Yes	No	Partially	Yes	7.0
[40]	Yes	Yes	Partially	Yes	Yes	Yes	No	No	Yes	6.5
[41]	Partially	Yes	Partially	Yes	Yes	Yes	Partially	No	Yes	6.5
[42]	Yes	Yes	Partially	Yes	Yes	Yes	Partially	Partially	Yes	7.5
[43]	Yes	Yes	Partially	Yes	Partially	Yes	Partially	Partially	Yes	8.0
[44]	Yes	Partially	Partially	Yes	Yes	Yes	No	Partially	Yes	6.5
[45]	Yes	Yes	Yes	Yes	Yes	Yes	No	No	Yes	7.0
[46]	Yes	Yes	Partially	Yes	Yes	Partially	Partially	Yes	Yes	7.5
[47]	Yes	Yes	Yes	Yes	Yes	Yes	No	Yes	Yes	8.0
[48]	Yes	Yes	No	Yes	Yes	Yes	Partially	Partially	Yes	7.0
[49]	Yes	Yes	Yes	Yes	Partially	Partially	No	Partially	Yes	6.5
[50]	Yes	Yes	Yes	Yes	Yes	Yes	No	Yes	Yes	8.0
[51]	Partially	Yes	Partially	Yes	Yes	Partially	No	Yes	Yes	6.5
[52]	Yes	Yes	Yes	Yes	Yes	Yes	No	Partially	Yes	7.5
[53]	Yes	Yes	Yes	Yes	Partially	Yes	No	No	Yes	6.5
[54]	Yes	Yes	Partially	Yes	Partially	Partially	Partially	Partially	Yes	6.5
[55]	Yes	Yes	Yes	Yes	Yes	Partially	No	No	Yes	6.5
[56]	Yes	Yes	Partially	Yes	Yes	Yes	Yes	Yes	Yes	8.5
[57]	Yes	Yes	Yes	Yes	Yes	Yes	No	No	Yes	7.0
[58]	Yes	Yes	Yes	Yes	Partially	Yes	No	Yes	Yes	7.5
[59]	Partially	Yes	Yes	Yes	Partially	Partially	No	Yes	Yes	6.5
[60]	Yes	Yes	Yes	Yes	Partially	No	No	Yes	Yes	6.5
[61]	Yes	Yes	Partially	Yes	Yes	Yes	No	Yes	Yes	7.5
[62]	Yes	Yes	Partially	Yes	Partially	Partially	Partially	Yes	Yes	7.0
[63]	Yes	Yes	Partially	Yes	Partially	Yes	Partially	No	Yes	6.5
[64]	Yes	Partially	Yes	Yes	Partially	Yes	No	Yes	Yes	7.0
[65]	Yes	Yes	Partially	Yes	Partially	No	Partially	Yes	Yes	6.5
[66]	Yes	Yes	Yes	Yes	Partially	Partially	No	Yes	Yes	7.0
[67]	Yes	Partially	Partially	Yes	Yes	Yes	No	Yes	Yes	7.0
[68]	Yes	Yes	Yes	Yes	Partially	Partially	Yes	No	Yes	7.0
[69]	Yes	Yes	Partially	Yes	Partially	Yes	No	Yes	Yes	7.0
[70]	Yes	Yes	Yes	Yes	Partially	Yes	No	Partially	Yes	7.0
[71]	Yes	Yes	Yes	Yes	Yes	Yes	Partially	Partially	Yes	8.0
[72]	Yes	Yes	Partially	Yes	Partially	Partially	No	Yes	Yes	6.5
[73]	Yes	Partially	Partially	Yes	Yes	Yes	No	Yes	Yes	7.0
[74]	Partially	Yes	No	Yes	Yes	Yes	No	Yes	Yes	6.5
[75]	Yes	Yes	No	Yes	Partially	Yes	Yes	No	Yes	6.5
[76]	Yes	Yes	Yes	Yes	Yes	Yes	Partially	No	Yes	7.5
[77]	Yes	Yes	Yes	Yes	Yes	Yes	Yes	No	Yes	8.0
[78]	Yes	Yes	Yes	Yes	Partially	Yes	No	No	Yes	6.5
[79]	Partially	Yes	No	Yes	Yes	Yes	No	Yes	Yes	6.5
[80]	Yes	Yes	Partially	Yes	Yes	Yes	No	No	Yes	6.5
[81]	Yes	Yes	Partially	Yes	Yes	Yes	Partially	Yes	Yes	8.0

**Table 3:** All robotics competitions found and their respective references.

Robotics Competitions	References Where It Was Found
Robot World Cup Initiative—RoboCup	[3,4,6,8,10,17,24,26,28,29,32,35,36,37,38,39,43,44,47,48,49,51,59,65,66,67,76,79,81]
World Robot Summit (WRS)	[59,75,80]
Agile Robotics for Industrial Applications Competition (ARIAC)	[77]
Mohamed Bin Zayed International Robotics Competition (MBZIRC)	[1]
e-Yantra Robotics Competition (eYRC)	[34,45,69,71,78]
Micromouse	[3,60,70]
Robot@Factory competition/Robot@Factory Lite competition	[5,40,41,46,63,67]
MicroFactory	[41,46]
RoboParty	[70]
Portuguese Robotics Open	[70]
Bot Olympics	[70]
Firefigher Robot	[70]
Robot Competitions Kick Innovation in Cognitive Systems and Robotics (RoCKIn) Competition	[36,37,44,48,59,67]
Student Autonomous Underwater Vehicles Challenge-Europe (SAUC-E)	[2,39,53,62,67,73]
An Outdoor Robotics Challenge for Land, Sea and Air (EURATHLON)	[2,33,39,44,53,54,62,67,73]
The European Robotics League (ERL)/ERL Emergency	[53,54,59,66,67,74]
DARPA Robotics Challenge	[3,4,8,27,28,32,33,35,36,39,44,53,59,62,65,66]
FIRA HuroCup	[24,65]
IEEE Humanoid application challenge	[65]
Amazon Picking Challenge (APC)	[59]
European Robotics Challenge (EuRoC)	[59]
International Aerial Robotics Competition (IARC)	[28,49,56]
IROS Robotic Grasping and Manipulation Competition	[59]
Balam Robot Competition	[61]
National Robotics Competition in Guatemala	[61,72]
Freescale Cup	[61]
FIRST Lego League (FLL)/FIRST Robotics Competition/FIRST Technology Challenge	[3,4,8,9,10,18,22,29,32,39,42,43,50,52,55,58,72]
4-H Robotics	[50]
Mississippi BEST (MSBEST) Robotics	[50]
VEX Robotics Competition	[32,39,64]
World Robot Olympiad (WRO)	[4,8,10,29,43,55]
Brazilian Robotics Olympiad	[42]
European Land Robot Trial (ELROB) competition	[27,28,33,39,44,62]
SpaceBot Cup—a German robotics competition	[44]
BotBall	[4,8,10,29]
Australian Multi Autonomous Ground-Robotic International Challenge (MAGIC)	[27,37]
Defense Advance Research Projects Agency Robotics Challenge	[37]
National Instruments Autonomous Robotics Competition (NIARC)	[32]
RoboWaiter	[25,31]
Trinity College Fire-Fighting Home Robot Contest (TCFFHRC)	[25]
British MoD Grand Challenge	[27]
Robotic Day Like Follower Competition	[68]
EUROBOT	[20,30]
Cybertech	[20,21]
CiberMouse@RTSS08	[23]
Indoor Aerial Robot Competition	[19]
AAAI Mobile Robot Competition and Exhibition	[66]
“Schüler bauen Roboter” program	[57]
Urban Search and Rescue (USAR)	[33,39]
Humanitarian Robotics and Automation Technology Challenge(HRATC)	[35]
Robomagellan	[7]
RoboParty	[3]
Latin American IEEE Robotics Competition	[17]
Robosub	[73]
MATE ROV	[73]
Microtransat Challenge	[73]
SailBot	[73]
Underwater robot competition	[73]
RoboBoat	[73]
World Robotics Sailing Championship	[73]
SeaPerch	[73]
Students Autonomous Underwater Vehicle (SAVe)	[73]
Oceanology International (OI) China	[73]
Maritime RobotX Challenge	[73]
Shell Ocean Discovery XPRIZE	[73]
Virtual RobotX	[73]
METRICS	[73]

**Table 4:** Description of robotics competitions taken from the data extraction phase.

Robotics Competitions		
Agile Robotics for Industrial Applications Competition (ARIAC)	Description	It is an annual competition organized by the NIST (National Institute of Standards and Technology) since 2017. The main goal is to test the agility of industrial robot systems and to enable industrial robots on shop floors to be more productive, autonomous and to require less time from shop floor workers [77].
	Where it takes place	This topic was not found in the papers.
	Target public	Researches to practitioners.
	Challenges and activities	Participants needs to implement a robot control system for a robot to overcome agility challenges in a simulated environment. The robot needs to realize kitting tasks, building the kits by picking up all the required items, which can be found on shelves, on the conveyor belt or in bins.
	Technologies applied	Gazebo (open source robotics simulation environment) and ROS—Robot Operating System.
	Application area	This kind of competition can contribute to the development of robotics applications for the manufacturing industry, realizing repetitive tasks, decreasing the human errors and allowing robots to work in areas inaccessible for humans.
An Outdoor Robotics Challenge for Land, Sea and Air (EURATHLON)	Description	It is a competition funded by European Union and coordinated by the University of the West of England. The main aim of euRathlon was to propose teams from academia and industry real world challenges testing the intelligence and autonomy of outdoor robots in scenarios inspired by the 2011 Fukushima accident [2,33,39,53,54,62,73].
	Where it takes place	First land competition: 2013 in Berchtesgaden, Germany. Second sea competition: 2014 in La Spezia, Italy. The final euRathlon Grand Challenge (air, land and sea): 2015 in Piombino, Italy.
	Target public	Teams from academy, company and industry.
	Challenges and activities	Challenges for autonomous robots of different domains (air, sea and land) in scenarios inspired by the 2011 Fukushima accident. In 2013 Eurathlon coordinates a robotic competition based on land and on the next year based on sea. The third year is the Grand Challenge, where the robots of three domains (land, sea and air) needs to cooperate in order to achieve objectives in a scenario set up to simulate a nuclear power plant ravaged by a tsunami. The scenario was based on the Fukushima disaster. The Grand Challenge is composed of three missions: localizing two missing workers in the disaster area, surveying the area of disaster to identify dangerous leaks and finally closing valves inside the building and underwater to stem the leaks. Three days of the competition are to practice and the grand challenge is the last two days.
	Technologies applied	This topic is not discussed in the articles.
	Application area	This kind of competition can contribute to increase the state of the art on the air, land and sea autonomous robots to help in natural disasters.
Balam Robot Competition (BRC)	Description	It is a local robotics competition in Guatemala started in 2015. The main objective is to show that technology is not complicated and mathematics or science are not boring for students [61].
	Where it takes place	Outreach Department of Universidad Galileo.
	Target public	Students.
	Challenges and activities	They prepare six weeks having workshops of four hours per week. The main challenge of BRC 1.0 was to build a sumorobot, teams had to compete by rounds against other sumobots and those who remained inside the tatami where who remain as finalists. After various rounds a winner was determined.
	Technologies applied	3D printing.
	Application area	This competition can contribute to education.
Brazilian Robotics Olympiad (BRO)	Description	It was started in 2007, created by a team of several university professors with the mission of promoting robotics among brazilian students with or without previous knowledge of robotics, fostering their interest to engage in science, technology and engineering studies and carrers. The olympiad is fully free for participants, being annually organized by volunteers from several brazilian universities [42].
	Where it takes place	Brazil.
	Target public	Students.
	Challenges and activities	The activities are divided in two modalities, practical and theoretical. The theoretical exams are designed to give the knowledge and contextualization about robotics, six levels of written tests are prepared by the organizers and based on the age of students. This model allows students to realize that what they are learning at school can be applied to solve real world problems. The practical exams are based on RoboCup Junior—Rescue mission. There is a simulated disaster environment where teams of four participants must build a robot fully autonomous to rescue victims. The robot must follow a safe path, avoid debris, overcome gaps, go over a mountain, identify victims and rescue them, taking them to a safe place. The best teams are selected by the Brazilian RoboCup Committee to represent Brazil in the RoboCup Junior international competition.
	Technologies applied	Arduino kits.
	Application area	Robotics competition has been an exciting and motivational tool for helping students to learn how to solve real problems in a practical way. It has been a good contribution for education.
CiberMouse @RTSS08	Description	It is a robotics simulation competition [23].
	Where it takes place	The competition took place remotely.
	Target public	This topic was not found in the papers.
	Challenges and activities	The simulation system creates a virtual arena with obstacles, a starting grid, a target area and the bodies of the robots. The bodies are composed of a circular base and are equipped with sensor, actuator and command buttons. The participants must create a software which controls the movements of a team composed of five virtual robots.
	Technologies applied	Robot simulators.
	Application area	This topic was not found in the papers, but it can be concluded that this kind of competition can contribute to the development of virtual solutions, also for education and dissemination of technology areas through students.
Cybertech	Description	It is a robotic competition organized anually by the Universidad Politécnica de Madrid (UPM) started in 2001 [21].
	Where it takes place	Madrid, Spain.
	Target public	Undergraduate students from universities all around the world.
	Challenges and activities	The students have to design and build a robot that participates in different events. The events include: Maze event (robot have to get out of a maze in a minimum time), line-following event (robot must follow a black line over a white back-ground), solar cars event (participants have to build an autonomous device that should be able to move inside a circuit being propelled just by solar energy), simulated robots event (participants have to develop a computer program to control a virtual robot that moves in a simulated maze) and bullfighting event (each team has to build a bullfighter robot that fights in the arena against a bull robot provided by the organization).
	Technologies applied	This topic was not discussed in the papers.
	Application area	This competition can contribute to the field of education, increasing the motivation of them towards engineering domains.
DARPA Robotics Competition	Description	It is an industrial competition focused on autonomous vehicles, which has prize money [3,4,27,33,35,39,53,59,62,66].
	Where it takes place	This topic was not found in the papers.
	Target public	This topic was not found in the papers.
	Challenges and activities	Includes several manipulation tasks. First editions have the objective of promote autonomous driving of road vehicles and then in the others editions promote humanoid robots able to execute complex tasks and in the last editions the focus was to promote the development of adaptive vehicles for military purposes. Started as a competition for autonomous cars and recently a simulated challenge focusing on humanoid robotics using Gazebo.
	Technologies applied	Gazebo.
	Application area	Robotic competitions are important in the learning process of youngsters and it is becoming more and more usual in schools and universities last few years. The competitions can contribute to several areas, like industry, society, search, but one of the most are benefited has been education.
e-Yantra Robotics Competition (eYRC)	Description	It is an annual competition organized by e-Yantra and hosted at IIT Bombay. The objective is to teach robotics concepts to the college students using a Project Based Learning (PBL). The competition is totally online [34,45,69,71,78].
	Where it takes place	Indian Institute of Technology Bombay—India.
	Target public	College students of Indian Institute of Technology Bombay.
	Challenges and activities	The competition is divided in different stages. Firstly, there is a preliminary test where participants answer questions related to aptitude, programming and electronics knowledge. In Stage 2 the participants combine the software and hardware parts to find the best solution, it also involves hardware testing, video and code submission. Each stage is subdivided into small tasks. In 2018 it was introduced a theme called “Thirsty Crow”, which aims to teach “Marker Based Augmented Reality”, for the first time. The teams need to build a robot (called Crow) capable of autonomously following the line and pick up the magnetic pebbles and drops them at the water pitcher marker. They also have to design and construct a 3D model of pebbles, water pitcher and Crow in Blender. They also have to write a python script related to the augmented reality part.
	Technologies applied	Marker based augmented reality using open source python libraries such as OpenCV and OpenGL; 3D modeling using blender software; ros; machine learning; image processing; microcontroller programming.
	Application area	This kind of competition can contribute to education, increasing the students’ interest in STEAM areas and robotics.
EUROBOT competition	Description	It is an international amateur robotics contest, organized by the Eurobot Association and founded in May 2004, but the contest was introduced already in 1998 [20,30].
	Where it takes place	Annually somewhere in Europe.
	Target public	Young engineering students.
	Challenges and activities	During a match, two opponents robots are competing on the table for 90 seconds, each robot is performing tasks defined in the rules. The robots must be autonomous and a robot should not collide with other opponent, if this happens the team is disqualified. The winner is the robot that collect more points. In the Eurobot 2010 edition the robot must collect fruits and vegetables, represented by balls and cylinders. In the Eurobot 2011 edition two mobile robots must play a “chess up”, the game is played on a playing table of the usual Eurobot size.
	Technologies applied	3D printing.
	Application area	The main application area is education.
European Land Robot Trial (ELROB)	Description	It was founded in 2006 by the European Robotics Group and organized by the Fraunhofer Institute for Communication, Information Processing and Ergonomics [27,33,39].
	Where it takes place	Annually at changing locations throughout Europe.
	Target public	This topic was not found in the papers.
	Challenges and activities	The ELROB alternates between military and civilian and defines a variety of scenarios instead of only one single mission. These tasks include: security missions, convoying or reconnaissance by day and night. The team can choose between the alternative scenarios. The scenarios also include detection of objects and transportation, which can be carried out with a single vehicle or a convoy with at least two vehicles.
	Technologies applied	2D and 3D laser scanner, 3D Lidar sensor, cameras, GPS and inertial sensors.
	Application area	Provides an opportunity to exchange ideas, create solutions as well as a venue to evaluate and encourage state of the art research. This competition can be helpful for the members of teams because they are forced to work together in a determined time, contributing to education field.
European Robotics League (ERL)/ ERL Emergency	Description	It is a multidomain robotic competition funded by the European Union Horizon 2020 Programme, which is focused on two indoor robotics competitions (ERL Industrial and ERL Service Robots) and one outdoor robotic competition (ERL Emergency Robots). The 2017 ERL Emergency competitions require flying, land and marine robots acting together to survey the disaster [53,54,66,74].
	Where it takes place	Many countries over Europe.
	Target public	This topic is not discussed in the papers.
	Challenges and activities	The competition has a duration of 9 days and the robots has to perform tasks related to land, air and sea domains which emulate real-world situations inspired by the 2011 Fukushima accident. The missions include: Mission A: Search for missing workers. Mission B: Reconnaissance and environmental survey. Mission C: Pipe inspection and stemming the leak. Robots have to work in a catastrophic scenario. From a starting point, the vehicle had to submerge, pass through the gate and it was then required to perform different tasks without resurfacing. The tasks include inspecting and mapping the area and the objects of interest, identifying mission targets, such as the leaking pipe and the missing worker.
	Technologies applied	Cloud resource and 4G connection.
	Application area	This kind of competition can contribute to increase the state of the art on the air, land and sea autonomous robots to help in natural disasters.
FIRA HuroCup	Description	It is a multi-event robot athletic competition intended to encourage breath in humanoid performance [24,65].
	Where it takes place	First edition in Seoul, Korea.
	Target public	This topic was not found in the papers.
	Challenges and activities	HuroCup is part of the FIRA international robotic competition and consists of robot dash, penalty kicks, lift and carry, basketball, weightlifting, climbing wall and obstacle run, the robot with the best score over all events is the winner.
	Technologies applied	This topic was not found in the papers.
	Application area	This topic was not found in the papers.
FIRST Robotics Competition/ FIRST Lego League (FLL)	Description	It is an international competition which began in 1998 as a joint effort between the FIRST (For Inspiration and Recognition of Science and Technology) Organization and the LEGO Group to introduce robotics to students. The competition has a duration of six weeks. The Lego League is designed for young ages [3,4,9,18,22,29,50,52,58].
	Where it takes place	In different countries around the world.
	Target public	High school and university students, engineers, technicians, business, leaders and concerned citizens. Lego League: students from 9 to 14 years old.
	Challenges and activities	Teams design and build tele-operated mobile robots to achieve a variety of tasks. In the Lego League they have to use LEGO kits to work on an authentic scientific-themed challenge, the themes include climate change, senior solutions, food safety, medicine, moving across a field, climbing ramps, hanging from bars and placing objects in goals. Each year, there is a new theme. The tasks first allow students to connect what they learn about robotics to what they could do in the face of real-world challenges and second, authentic tasks and plausible scenarios are structured to motivate students to overcome potential challenges in learning robotics.
	Technologies applied	LEGO Mindstorms, LabView software, sensors.
	Application area	This competition can connect students with professionals, enable them to solve real-world problems and develop 21st century skills. Robotic competitions have been a good tool for education because it aids universities to teach a variety of multidisciplinary engineering topics including design, programming and mechatronics.
Humanitarian Robotics and Automation Technology Challenge (HRATC)	Description	It is a humanitarian demining international robotics competition, which the goal is to push boundaries of what technology can accomplish in this field. The first edition happened in 2014 [35].
	Where it takes place	The entire competition is performed remotely.
	Target public	This topic was not found in the papers.
	Challenges and activities	The competition is divided in three stages: The simulation stage: teams must focus on their ideas and to develop their algorithms. The teams have to focus on the actual problem, humanitarian demining. The field trials stage: in this stage each team will be able to run the software developed on the simulator on the actual robot, each team has 3 field trials, throughout 3 weeks. Competition day: each team is given two runs on a minefield using surrogate mines and false positives.
	Technologies applied	Gazebo simulator, TeamBots simulator, ROS and USARSim simulador.
	Application area	The main contribution of this competition is to increase the state of the art in the area of humanitarian demining.
IEEE Humanoid application challenge	Description	The 2019 theme was robot magic [65].
	Where it takes place	This topic was not found in the papers.
	Target public	This topic was not found in the papers.
	Challenges and activities	In the robot magic theme a humanoid robot can take on any role in a magic show.
	Technologies applied	OpenCV (image processing) and PocketSphinx (speech recognition).
	Application area	Provides opportunity for improving work in robotics and a large range of areas of artificial intelligence (vision, speech understanding, interacting with humans).
Indoor Aerial Robot Competition	Description	It was inaugurated in May 2005 with the objective to identify best design practices and gain insight on technical challenges facing the development of unmanned air vehicles [19].
	Where it takes place	Swarthmore College.
	Target public	This topic was not found in the paper.
	Challenges and activities	The tasks are based on line-following and teleoperation. The teams have to implement a line-following algorithm in real time which is invariant to changing lighting conditions. The points are based on how far the robots are able to travel.
	Technologies applied	This topic was not found in the paper.
	Application area	It has been a means of discovering the best practices to solve real world problems.
International Aerial Robotics Competition (IARC)	Description	It is an international competition focused on aerial robots [49,56].
	Where it takes place	This topic is not found in the papers.
	Target public	This topic is not found in the papers.
	Challenges and activities	In this competition the agent (aerial robot) is required to contact targets (ground vehicles) sequentially and drive them to a certain boundary to earn score. The agent robot needs to be fully autonomous and the game has a duration of 10 min. In the IARC mission 7 called “Shepherd mission”, there is a drone, 10 ground mobile robots and 4 mobile obstacles. First, the drone should be able to avoid collision with four mobile obstacles. Second, there are two ways to change the moving direction of each ground mobile robot. The final target of winning the competition is to drive at least 4 out of the 10 ground mobile robots across the green edge of the square arena within 10 min.
	Technologies applied	This topic is not found in the papers.
	Application area	This topic is not found in the papers.
Latin American IEEE Robotics Competition	Description	It is an annually competition organized by the Department of Electrical Engineering of the Universidad de Chile and by the IEEE region 9 [17].
	Where it takes place	The first was held in Santiago—Chile.
	Target public	Engineering students.
	Challenges and activities	The first competition (“beginners”) was aimed for students to work in robotics and was based on Lego MindStorms building blocks, the proposed challenge is to design and programming a robot that cross a simulated minefield. The second competition (“advanced”) is designed for experienced students’ groups and consists of crossing a soccer field with obstacles using any kind of legged robots, the robots could be designed by participants, or could be bought or even adapted.
	Technologies applied	LEGO MindStorms.
	Application area	The main contribution is for the area of education.
MicroFactory	Description	It is a robotic competition designed to be low-cost and easily implementable in a small space and it is based on the Portuguese competition called Robot@Factory [41,46].
	Where it takes place	This topic was not found in the papers.
	Target public	High school students and university undergraduate students.
	Challenges and activities	The challenges are similar to the Robot@factory challenges but the ground area and complexity is reduced and the scenario material were simplified. In MicroFactory there are just 3 rounds.
	Technologies applied	3D printing, Arduino, odometry and sensors.
	Application area	The main contribution of this competition is for education.
Micromouse	Description	It is one of the most popular competitions inside the context of mobile robots started in 1970s, being the first competition promoted by the IEEE. It is organized at the University of Trás-os-Montes e Alto Douro [3,60,70].
	Where it takes place	Editions are held worldwide.
	Target public	Students, researches and the general public.
	Challenges and activities	A small autonomous mobile robot put in an unknown labyrinth must be able to map it, look for the best possible route between the starting point and the goal and travel it in the shortest time. The challenge is not solving the maze but how fast the robot can do it.
	Technologies applied	Scanning and path planning algorithms, fFloodfill procedure, HIL simulator, self-localization using odometry and distance sensors.
	Application area	The Micromouse competition is an important tool for the education, increasing the young students’ interest in STEAM but to introduce other people to the field of robotics.
Mississippi BEST (MSBEST) robotics	Description	It is a competition which has a mission to inspire students to pursue careers in STEM areas through robotic design and competition. [50].
	Where it takes place	Mississipi—USA
	Target public	Middle and high school students.
	Challenges and activities	The challenge has a duration of six weeks. The participants are supplied with kits of material and they have to put those material together to build a robot, participants have to do a search about the competition theme for that particular year, realize a brainstorm with the ideas on how to design the robot to perform tasks related to the theme. All the students are required to submit their notebook, team demographics and consent forms.
	Technologies applied	This topic is not discussed in the papers.
	Application area	The main application area is education.
Mohamed Bin Zayed International Robotics Competition (MBZIRC)	Description	It is an international robotics competition [1].
	Where it takes place	This topic was not found in the papers.
	Target public	This topic was not found in the papers.
	Challenges and activities	The Challenge 1 of the MBZIRC competition consists of aerial drone interception scenario. First, there are fixed balloons randomly around the arena and the autonomous aerial system must automatically detect, get close and blow up. Second, another autonomous aerial system should capture a ball that is suspended from another drone that flies at high speed on a variety trajectory. All these tasks must be performed autonomously.
	Technologies applied	Time-of-flight cameras, machine learning, computer vision and Kalman filters.
	Application area	This competition can contribute to increase the state of the art in autonomous vehicles and drones, which has been attracting a lot of attention, for example, for urban air mobility (UAM).
National Instruments Autonomous Robotics Competition (NIARC)	Description	It is a competition started in 2012 and its focus on fully autonomous robots to complete a given theme challenge. These themes have included search and rescue, mining and agriculture [32].
	Where it takes place	Universities across Australia and New Zealand.
	Target public	Students.
	Challenges and activities	In NIARC 2012 the theme was search and rescue, where teams have to develop a robot to navigate a grid based maze environment. The objective is the robot navigate in unknown maze and differentiate the victims. NIARC’s 2013 theme was the mining industry, where the objective was the robot navigate to the mining area through the unknown entrances and differentiate the desired gold cubes and undesired grey rubble cubes. NIARC’s 2014 theme was the agriculture industry and the objective is the robot navigate accurately to known but unmarked seeding areas to plant seeds.
	Technologies applied	Real time control, FPGA, LabView.
	Application area	Studies have shown the benefits of using robotics competitions to generate interest and motivation in studying engineering for high school students and general public. Besides that it can also contribute to develop the ability of the teams to work together in multidisciplinary work.
RoboCup	Description	It is an international competition which runs by the RoboCup Federation and the goal is: “By the year 2050, a team of fully-autonomous humanoid robot soccer players shall win a soccer game, complying with the official FIFA rules, against the winner of the most recent World Cup of Human Soccer” [4,6,8,10,17,26,28,29,36,43,47,48,49,51,59,76,79,81].
	Where it takes place	The first edition took place in Nagoya, Japan and now the competition take place in many countries all over the world annually.
	Target public	Since senior participants like researchers and university students to hobbyists, high school, primary and secondary students.
	Challenges and activities	There are different leagues: Junior: for young students. There are three ages categories and three leagues being them soccer, rescue and dance. This league keeps the same activities over the years to help students improving their solutions. Soccer game: teams with autonomous robots compete each other in a soccer game; Search and rescue: robots that can assist first responders in mitigating a disaster such as an earth-quake or an accident in an industrial environment; Home: service robots to realize household activities; Work: defines nine challenges inspired by industrial mobile manipulation and transport tasks; Logistics League: groups of three robots have to plan, execute and optimize the material flow and deliver products according to dynamic orders in a simplified factory; Humanoid Challenge: humanoid robots compete in three events: walking, penalty kicks and a free demonstration; Simulation 2D and 3D: two teams of eleven software agents compete against each other on a simulated soccer pitch; Small size: semi-autonomous soccer robots (diameter of 18 cm and height of up to 15 cm); Middle size: slow driving robots (that drive up to 4m/s) on small soccer fields enclosed by walls; Standard Platform: soccer game in which all teams compete with identical robots.
	Technologies applied	Sensor, actuators, AI solutions, machine learning, multi-agent coordination, ROS, SLAM, image processing, wireless standard communication interfaces, LEGO Mindstorms, object recognition, speech recognition and gesture recognition.
	Application area	How the competition includes several leagues the application area can be more than one, increase the state of the art in the leagues areas, attract more students to STEM concepts contributing to education through RoboCup Junior and also increase the development of solutions for industry, daily life and natural disasters like RoboCup Home, Logistics League and Search and Rescue.
Robomagellan	Description	It is an outdoor navigation competition hosted by RoboGames [7].
	Where it takes place	This topic is not discussed in the papers.
	Target public	This topic is not discussed in the papers.
	Challenges and activities	The competition requires the robot moves in an unconstrained and unstructured real-world outdoor environment with different obstacles.
	Technologies applied	ROS, FSM and extended Kalman filter (EKF).
	Application area	The robotic competition contributes with the advances in the field of robotics.
Roboparty	Description	It is an educational robotic event with a duration of three non-stop days [3,70].
	Where it takes place	Universidade do Minho, Guimarães, Portugal
	Target public	School-age children.
	Challenges and activities	The students learn by experience how to build the Bot’n Roll robotic platform (mechanics, soldering electronics components and assembling the parts). Then, three challenges are executed to test their robots and the developed algorithms.
	Technologies applied	This topic is not discussed in the papers.
	Application area	The main application is education.
Robosub	Description	It is the first AUV competition and the first edition was in 1997. Currently it is the most popular competition in the AUV world and every year the competition has a different theme [73].
	Where it takes place	USA.
	Target public	Students (high school and university)
	Challenges and activities	The AUV mission consists of passing a gate, touching a buoy, dropping and retrieving objects and launching a plastic marker inside a target hole.
	Technologies applied	This topic was not found in the papers.
	Application area	The main application area is education.
RoboWaiter	Description	The first robot competition in the area of assistive robotics, it is conducted in conjunction with the annual international Trinity College Fire-Fighting Home Robot Contest (TCFFHRC) in 2009. The vision of the competition is: bringing people with disabilities as clients of RoboWaiter design and Integrating the RoboWaiter project in a robotics course [25,31].
	Where it takes place	Hartford, Connecticut
	Target public	Traditional participants are students, hobbyists and engineers.
	Challenges and activities	Each robot has three runs and must navigate autonomously from its home position to a scale-model refrigerator, pick up a plate of food from a shelf, navigate to the table where a person with mobility impairment is sitting, places the plate on it and return to home position. Robots must avoid collisions with obstacles (sink, chair and elderly person).
	Technologies applied	This topic was not found in the paper.
	Application area	Development of solutions based on assistive robots to help people with disabilities realizes activities more easily. Other application area can be the education once the competition encourages students to STEAM areas.
Robot Competitions Kick Innovation in Cognitive Systems and Robotics (RoCKIn) Competition	Description	It aims to provide tools for benchmarking to the robotics community by designing and setting up competitions that increase scientific and technological knowledge. It is inspired by the RoboCup [44,48,59].
	Where it takes place	The first was held in Toulouse, France in 2014. The final was held in Lisbon, Portugal in 2015.
	Target public	This topic was not found in the papers.
	Challenges and activities	RoCKIn@Work: there is a medium sized factory which produces small to medium sized lots of mechanical parts and assembled mechanical products, the robots must try to optimize its production process to meet the increasing demands of their customers. RoCKIn@Home: the robots must assist a person and supporter quality life, it is based in an apartment with all common household items like windows, doors, furniture and decorations.
	Technologies applied	This topic was not found in the papers.
	Application area	Robotics competitions have been a good way to promote comparison of different algorithms and systems, allowing for the replication of their results. Robotics competitions also contributes for promoting education and research to push the field forward.
Robotic Day Line Follower Competition	Description	It is an annual competition which has been occurring during the last 15 years and it is growing every year. This competition used topics that can be used as benchmark, comparing different performances [68].
	Where it takes place	Prague, Czech Republic.
	Target public	This topic is not discussed in the article.
	Challenges and activities	The participants’ robots must to run in a way and follow a black line. They need to pass obstacles and the robot that complete the route in the shortest time qualify for the finale. In the final round the races are held on a knock-out.
	Technologies applied	Time of flight distance sensor and computer vision.
	Application area	In this competition context it is possible to apply the activities in multidisciplinary approach contributing to education. It can also have an importance in research and development, because the outcomes can be applied to solve real world problems, for example, in manufacturing and service robots.
Robot@Factory Robot@Factory Lite	Description	It is an annually competition started in 2011 recently included in Robotica, the main Portuguese Robotics Competition. Robot@Factory Lite is a simplified version. The goal is to stimulate students and researchers to develop solutions to the challenges proposed by them [5,40,41,46,63,67].
	Where it takes place	Portugal.
	Target public	Secondary school and universities students.
	Challenges and activities	The competition deal with the problem of the transportation of materials inside a factory. The main idea is an AGV organize the materials in warehouses with processing machines. There are four warehouses with two machines, the robot must deliver the parts in their correct locations. The AGV must be fully autonomous. The competition is divided in three days, each day has a round.
	Technologies applied	SimTwo simulator, which is provided by the competition and hardware in the loop (HIL), a software where the competitors insert their microcontroller in the loop of the simulation.
	Application area	The main application area is education.
“Schüler bauen Roboter” program	Description	“Schüler bauen Roboter” is a German project that brings together schools and universities [57].
	Where it takes place	Technical University of Munich, Germany.
	Target public	Target group is 14 to 18 years old high school students.
	Challenges and activities	In the first school year the students can build a robot that solve a given task and at the end of the year, the different groups can compete against each other. Usually the competition starts in September, when the school year begin.
	Technologies applied	This topic was not found in the paper.
	Application area	The main application area is education, once the competition was created to take place inside a university to help them to encourage students in the STEM areas and get skills in programming, electronics, robotics, etc.
SICK robot day	Description	It is a bi-annual competition hosted by SICK AG, a producer of sensor systems [38].
	Where it takes place	Waldkirch, Germany.
	Target public	This topic was not found in the papers.
	Challenges and activities	The robots must navigate autonomously and avoid obstacles and collision with other robots. The goal is to deliver as many objects as possible, where each correctly delivered object was awarded one point and each erroneous delivery one penalty point. With a limit time of 10 min, each robot had to alternately collect labelled objects at filling stations and transport them to delivery stations based on the object label.
	Technologies applied	This topic was not found in the papers.
	Application area	This topic was not found in the papers.
SpaceBot Cup	Description	It is a German robotics competition started in 2013. The second edition occurred in 2015. The main of the competition is to accomplish (conclude, finish) this activity as autonomous as possible by means of unmanned vehicles. The focus is mobile manipulation for planetary exploration [44].
	Where it takes place	Germany.
	Target public	Universities, research institutes and subject matter experts (SMEs).
	Challenges and activities	SpaceBot has only one scenario which involves typical exploration tasks carried out on a planetary surface after landing on a planet. The robots have to locate and identify objects in a complex terrain. The target objects needs to be conveyed (transmitted) to a base station. The robots needs to be autonomous, communication was only allowed through a shaped network connection that imposed restrictions on the ports used. The tasks to be accomplished were: explore and map the terrain, find artificial objects, collect the two objects and bring them to a third and finally return to landing site.
	Technologies applied	This topic was not found in the paper.
	Application area	This topic was not found in the paper.
Student Autonomous Underwater Vehicle Challenge- Europe (SAUC-E)	Description	It is the first underwater robotics competition in Europe. SAUC-E started in 2006 in the UK and then has been hosted by CMRE since 2010, the main goals are: advance the state of art of AUV, promote creative environment among researches, get closer contact between the university teams and companies invited to participate [2,53,62,73].
	Where it takes place	In many countries around Europe.
	Target public	University students.
	Challenges and activities	The typical tasks includes passing through the gate, mapping and inspecting an underwater pipeline structure, localizing on the seafloor a pinger that emit an acoustic wave and localizing underwater buoys and objects. The task must be done totally autonomous by the robot.
	Technologies applied	This topic was not found in the papers.
	Application area	The main application area is education.
VEX Robotics Competition	Description	It is a competition which aim to engages participants from elementary through university students in learning about STEM concepts. This competition was launched in 2005 and today it is one of the largest extracurricular robotics program in the world [32,39,64].
	Where it takes place	The place is not discussed in the articles.
	Target public	Middle school, high school and university students.
	Challenges and activities	This topic was not found in the papers.
	Technologies applied	This topic was not found in the papers.
	Application area	The main application area is education.
World Robot Olympiad (WRO)	Description	It was founded in 2004 and the initial mission is: to bring together young people all over the world to develop their creativity, design and problem solving skills through challenging and educational robot competitions and activities [4,55].
	Where it takes place	This topic is not discussed in the papers.
	Target public	Students.
	Challenges and activities	There are three categories: regular category (the robots complete tasks and it is open for 13–15 years), open category (build a robot model) and football category (teams build two robots who compete against another team’s robots in a robot football match). The theme of the 2018 edition was “Precision Farming” requires the students to design a robot which is able to plant different coloured seedlings in the corresponding farm areas.
	Technologies applied	LEGO Mindstorms.
	Application area	The main application area is education.
World Robot Summit (WRS)	Description	It is an international competition started in October 2018, organized by the Japanese government to accelerate research and development of robots in the areas of daily life, society and industry in order to promote a world where humans and robots successfully live and work together [59,75].
	Where it takes place	Tokyo, Japan
	Target public	This topic was not found in the papers.
	Challenges and activities	The leagues: rescue, service and assembly. Service: the tidy up here task consists of moving objects from incorrect positions to the right positions. There are four rooms, children, dining, kitchen and a living room. There are two types of objects, 45 known units and 10 unknown units. Assembly: aims to develop robots to allow the assembly of complex systems with varied products. There some tasks like task board, kitting, assembly and surprise assembly.
	Technologies applied	Simultaneous localization and mapping (vSLAM), vision system (TensorFlow).
	Application area	The competition can contribute to development of novel solutions, providing a benchmark for robot assistance, not only for disabled people but also for elderly people and healthy people, by assisting daily housekeeping tasks. Can also contribute to the manufacture industry developing solution to assembly products.

**Table 5:** Research topics associated with the mobile robotics competitions and related papers.

Robotics Competitions	Simulation	Control	Localization and/or Mapping	Obstacle Avoidance	Prototyping	Computer Vision	Navigation	Artificial Intelligence
ARIAC	[77]	[77]	[77]	[77]		[77]	[77]	
EURATHLON		[2,33,39,53,54,62,73]	[2,33,39,53,54,62,73]	[2,33,39,53,54,62,73]		[2,33,39,53,54,62,73]	[2,33,39,53,54,62,73]	
BRC		[61]			[61]			
BRO	[42]	[42]	[42]	[42]		[42]	[42]	
Cibermouse	[23]	[23]		[23]			[23]	
Cybertech	[21]	[21]			[21]		[21]	
DARPA	[3,4,27,33,39,53,59,62,66]	[3,27,33,35,39,53,59,62,66]	[3,27,33,35,39,53,59,62,66]		[3,27,33,35,39,53,59,62,66]	[3,27,33,35,39,53,59,62,66]	[3,27,33,35,39,53,59,62,66]	
eYRC					[34,45,69,71,78]	[34,45,69,71,78]	[34,45,69,71,78]	[34,45,69,71,78]
EUROBOT		[20,30]	[20,30]	[20,30]	[20,30]	[30]	[20,30]	
ELROB			[27,33,39]			[27,33,39]	[27,33,39]	
ERL		[53,54,66,74]	[53,54,66,74]		[53,54,66,74]	[53,54,66]	[53,54,66,74]	
FIRA HuroCup		[24,65]			[24]	[65]		[65]
FIRST					[3,4,9,18,22,29,50,52,58]	[3,4,9,18,29,50,52,58]	[3,4,9,18,29,50,52,58]	
HRATC	[35]	[35]						
IEEE Humanoid application challenge		[65]				[65]		[65]
Indoor Aerial Robot Competition		[19]					[19]	
IARC		[56]						[49]
Latin American IEEE				[17]	[17]		[17]	
Microfactory	[41]	[41,46]			[41,46]			
Micromouse	[3,60]	[3,60]			[3,60]		[3,70]	
MSBEST		[50]			[50]			
MBZIRC		[1]				[1]	[1]	[1]
NIARC		[32]				[32]	[32]	
RoboCup	[4,6,8,17,29,43,47,48]	[4,6,8,10,17,26,28,29,36,43,47,48,59]	[4,6,8,17,26,28,29,36,43,47,48]	[4,6,8,17,28,29,43,47,48]	[4,6,8,10,17,29,36,43,47,48]	[4,6,8,17,26,28,29,43,47,48]	[4,6,8,17,29,43,47,48]	[4,6,8,17,26,28,29,43,47,48,49,51,76,79,81]
Robomagellan		[7]	[7]	[7]			[7]	
Roboparty		[3,70]			[3,70]			
Robosub		[73]				[73]	[73]	
RoboWaiter		[25,31]	[25,31]	[25,31]	[25,31]	[25,31]	[25,31]	
RoCKIn		[44,48,59]	[44,48,59]				[44,48,59]	
Robotic Day		[68]		[68]		[68]	[68]	
Robot@ Factory	[5,40,41,46,63,67]	[5,40,41,46,63,67]	[5,40,41,46,63,67]		[5,41,46,63,67]	[41,46,63,67]	[5,40]	
“Schüler bauen Roboter”		[57]			[57]			
SICK robot day				[38]		[38]	[38]	
SpaceBot Cup		[44]	[44]	[44]		[44]	[44]	
SAUC-E		[2,53,62,73]	[2,53,62,73]			[2,53,62,73]	[2,53,62,73]	
VEX		[32,39,64]			[32,39,64]			
WRO		[4,55]			[4,55]			
WRS		[59,75]	[59,75]			[59,75]	[59,75]	

**Table 6:** Summary of the research questions answers.

Research Questions	Answers
RQ1: What type of mobile robotics competitions exist in the last few decades and with what aim?	Educational: attracting students to STEM areas encouraging them to enter in technologies careers. Industry: manufacturing and logistics. Domestic: assist people in household activities, especially those with disabilities. Sports: amusement for young students with focus on education. Search and rescue: creation of technologies to assist in natural disaster. State of the art: push the state of the art in a specific field like autonomous vehicles, drones and underwater robots.
RQ2: Where do the mobile robotics competitions take place currently and who is their target public?	Most take place in many countries all over the world. Some educational competitions usually take place inside a university or school in a specific region. The target public vary a lot, from young students until professionals and engineers.
RQ3: What type of robotics challenges are addressed by the mobile robotics competitions?	Education: include tasks in which the participants have to build a robot to perform some activity or sometimes just program the robot to realize a specific task, for example follow a line, dance, games, etc. Industry: the challenges vary between transport products and logistics tasks, in which the robot must navigate, avoid obstacles, pick up items, etc. Domestic: usually the robots have to help people with household tasks inside an environment that simulates a house. Some tasks include organizing items, opening the refrigerator, picking up a plate of food, etc. Search and rescue: tasks like climbing ramps, walking on dirty terrain with poor visibility, looking for missing people, pipe inspection, etc. Besides that this kind of competition can include underwater fields, buildings, small places, indoor and outdoor environments. Soccer: the soccer challenges usually includes teams of autonomous robots to compete against each other.
RQ4: What type of technologies are used in mobile robotics competitions?	Machine Learning, computer vision, localization and mapping (SLAM), speech, object and gesture recognition, real-time control, 3D printing, ROS, HIL, SimTwo, Gazebo, OpenCV, TensorFlow, LEGO Mindstorms, etc.
RQ5: What is the final application area of the mobile robotics competitions?	Education, industry, daily life, household tasks, search and rescue in disaster scenarios and amusement.
RQ6: How have these competitions been contributing positively to education?	Dissemination of technology and STEM concepts through young students, encouraging them to pursue a career in these fields, developing skills in programming, electronics, robotics, working in a team, increasing the motivation and besides that assisting universities to teach multidisciplinary domains.

## Data Availability

The dataset containing information about the mobile robotics competitions and related URLs to each paper in which the competition is cited, can also be accessed in Zenodo repository through the link: https://doi.org/10.5281/zenodo.6337324 (accessed on 8 March 2022).

## Abbreviations

The following abbreviations are used in this manuscript:

PRISMA	Preferred Reporting Items for Systematic Reviews and Meta-Analyses 6
STEM	Science, Technology, Engineering and Mathematics
STEAM	Science, Technology, Engineering, Arts and Mathematics
AGV	Automated Guided Vehicle
AUV	Autonomous Underwater Vehicle
FIRST	For Inspiration and Recognition of Science and Technology
SLR	Systematic Literature Review
PICOC	Population, Intervention, Comparison, Outcomes, Context
RQ	Research Question
QQ	Quality Question
DQ	Data Question
WRS	World Robot Summit
ARIAC	Agile Robotics for Industrial Applications Competition
MBZIRC	Mohamed Bin Zayed International Robotics Competition
eYRC	e-Yantra Robotics Competition
RoCKIn	Robot Competitions Kick Innovation in Cognitive Systems and Robotics
SAUC-E	Student Autonomous Underwater Vehicles Challenge-Europe
ERL	European Robotics League
APC	Amazon Picking Challenge
EuRoC	European Robotics Challenge
IARC	International Aerial Robotics Competition
FLL	FIRST Lego League
MSBEST	Mississippi BEST
WRO	World Robot Olympiad
BRC	Balam Robot Competition
ELROB	European Land Robot Trial
MAGIC	Australian Multi Autonomous Ground-Robotic International Challenge
NIARC	National Instruments Autonomous Robotics Competition
TCFFHRC	Trinity College Fire-Fighting Home Robot Contest
USAR	Urban Search and Rescue
HRATC	Humanitarian Robotics and Automation Technology Challenge
SAVe	Students Autonomous Underwater Vehicle
OI	Oceanology International
ROS	Robot Operating System
SLAM	Simultaneous Localization and Mapping
AI	Artificial Intelligence
NIST	National Institute of Standards and Technology
vSLAM	Visual Simultaneous Localization and Mapping
UAM	Urban Air Mobility
PBL	Project Based Learning
HIL	Hardware in the Loop
FSM	Finite State Machine
EKF	Extended Kalman Filter
FPGA	Field-programmable Gate Array
GPS	Global Positioning System
UPM	Universidad Politécnica de Madrid
USARSim	Unified System for Automation and Robot Simulation
USA	United States of America
